# The Value of Current *Ante Mortem* Meat Inspection and Food Chain Information of Dairy Cows in Relation to *Post Mortem* Findings and the Protection of Public Health: A Case for a More Risk-Based Meat Inspection

**DOI:** 10.3390/foods12030616

**Published:** 2023-02-01

**Authors:** Pieter Jacobs, Boyd Berends, Len Lipman

**Affiliations:** 1 Netherlands Food and Consumer Product Safety Authority (NVWA), P.O. Box 43006, 3540 AA Utrecht, The Netherlands; 2 Institute for Risk Assessment Sciences, Department of Population Health Science, Faculty Veterinary Medicine, Utrecht University, P.O. Box 80175, 3508 TD Utrecht, The Netherlands

**Keywords:** meat safety, meat inspection, risk-based, legislation, veterinarian, official control

## Abstract

In this study, the contribution of the *ante mortem* (AM) inspection and the food chain information (FCI) to ensuring meat safety and public health was investigated, by evaluating the slaughterhouse findings of 223,600 slaughtered dairy cows in the Netherlands. The outcome of this study was that the *ante mortem* (AM) and *post mortem* (PM) inspections have a substantial overlap, and that with regard to food safety and public health in over 99% of cases the PM could even be omitted on the basis of the AM. In this study, the data provided by the dairy farmers on the current FCI forms contributed little to nothing with regard to the outcomes of AM and PM inspection. It is concluded that current meat inspection procedures need an update and a more risk-based approach needs to be adopted. Regarding this, the AM inspection of dairy cattle should remain, because it plays an important role in ensuring food safety (e.g., by preventing contamination of the slaughter line by excessively dirty animals, or animals with abscesses), monitoring animal welfare and in detecting some important notifiable diseases. The PM inspection, however, could in many cases be omitted, provided there is a strict AM inspection complemented by a vastly improved (automated) way of obtaining reliable FCI.

## 1. Introduction

Current meat inspection was originally designed in Europe in the late 19th century and was almost entirely aimed at protecting the public’s health [1]. With increasing international trade, detecting notifiable animal diseases soon became another important goal. The most recent addition to its goals is monitoring animal welfare. For all of these reasons, all animals destined for slaughter are subjected to a brief clinical examination by an official veterinarian before they enter the slaughter line (i.e., the *ante mortem* inspection, AM) and a concise pathological–anatomical examination after most of the internal organs have been removed and made accessible for close inspection (i.e., the *post mortem* inspection, PM). These two examinations together determine (i) whether an animal may be slaughtered for human consumption, and (ii) if that slaughtered animal is fit for human consumption, so that its meat and edible by-products may indeed enter the human food chain. The way AM and PM are performed in the EU is currently laid down in the Official Controls Regulation EU 2017/625 and follows a strict protocol regardless of the age of the animals or any other factor that may influence the possible outcomes and value of these procedures [2,3,4].

However, the threats to public health that can be associated with the slaughter of animals have changed during the last century, whereas the system of meat inspection has remained basically the same. Therefore, it seems that current meat inspection procedures are no longer adequate in protecting public health, and there is a need for a more risk-based form of meat inspection [5]. 

At the time meat inspection was designed, virtually all zoonotic diseases that were of primary concern had distinct clinical signs and/or caused distinct macroscopic pathological–anatomical abnormalities, as, for example, was the case for tuberculosis, anthrax and cysticercosis [1,6,7]. Currently, these zoonotic conditions do not play a significant role in modern western countries or are no longer even considered to be a major health risk anymore, such as bovine cysticercosis [8]. The currently important human health hazards remain practically always undetected during AM and PM inspection. Examples of these are animals that contain residues of veterinary drugs or environmental contaminants, animals that are infected with *Toxoplasma gondii*, and animals that are fecal carriers of *Salmonella spp*., *E. coli* O157:H7 or Extended Spectrum Beta Lactamase (ESBL) producing *Enterobacteriaceae* [9,10,11,12,13,14].

Thus, the value of meat inspection with regard to its efficacy in protecting human and animal health in situations where animals are raised in modern systems of husbandry and provided with optimum health care may be seriously questioned [15]. Nowadays, the main function of the AM meat inspection appears to be (a) preventing the contamination of the slaughter line (e.g., by excessively dirty animals, or animals with an abscess), (b) monitoring animal welfare, and (c) acting as a last line of defense with regard to several notifiable animal diseases. *Post mortem* meat inspection, on the other hand, serves to detect abnormalities that are almost entirely related to food quality and on-farm (health) management issues [9,16,17,18].

This study was aimed at (a) assessing the value of the current EU meat inspection procedures with regard to the condemnation of whole carcasses declared ‘not suitable for human consumption’ (NHC) in the framework of the protection of public health, and (b) gauging whether data from official meat inspections—as a proof of principle—can potentially be used for determining which AM or PM procedures could, in a particular situation, be revised or even omitted (i.e., to transform our current system into a more flexible and risk-based one).

## 2. Materials and Methods

### 2.1. Population and Slaughterhouse

The slaughter of dairy cows was chosen as a test-case, because the slaughter of this group probably best resembles the situation for which meat inspection was originally designed. When compared to the slaughter of pigs or poultry, the number of animals from a farm sent to slaughter in one shipment is relatively low and the animals are also far more diverse with regard to the circumstances they were kept under, as well as their genetic make-up. Furthermore, dairy cows are currently the animals that, at the time of slaughter, display the largest variations in age and disease history [19].

For this study, the data from 223,600 animals that were slaughtered in 2014 and 2015 in the largest cattle slaughterhouse in the Netherlands were used. This slaughterhouse is considered to be representative for the entire Dutch situation, since the only difference with the other slaughterhouses was the scale of operations and not, for example, the type of breeds slaughtered or the regions from where the animals originated.

### 2.2. Data Sources and Management

The main data regarding the results of the AM and PM inspections came from the database for the Registration of Slaughter Findings (Registratie Slacht Gegevens, RSG) of the Netherlands Food and Consumer Product Safety Authority (NVWA) [19] and from the individual findings during the inspections of each animal as written by hand on official forms from the NVWA, also called “VOS forms” (Verzamelstaat Onderzoeksgegevens Slachtdieren, i.e., Summary Findings Meat Inspection) [20]. On these VOS forms, all relevant findings of the AM and PM inspection are briefly noted by the official veterinarians, including the final decision regarding the carcass and organs.

The Identification and Registration (I&R) data came from the official database for the registration of animals in the Netherlands. These data were needed for determining the location of the farms of origin, the age of the animals, their parity and their breed.

The individual Food Chain Information (FCI) forms were included in this study, when any relevant information was available. Food Chain Information is legally required for all animals to be slaughtered for human consumption (as laid down in EU regulation 853/2004, annex II, Section III) [5].

As a first step, all 223,600 VOS forms from 2014 and 2015 were—in a period of several months—thoroughly read. All the VOS forms of animals that were declared not suitable for human consumption (NHC) at the *post mortem* examination (PM) were used as a basis, and put in a spreadsheet together with the information about the AM inspection results, the information from the FCI forms, and information from the RSG and I&R databases. All data from 3933 individual NHC animals thus gathered were subsequently used for further analyses.

### 2.3. Definitions and Categorizations

The criteria determining whether an animal is suitable for human consumption are laid down in European legislation [4]. If an animal is declared ´NHC’ by the official veterinarian, Regulation EU 2017/625 considers it by definition an unacceptable risk for food safety and/or public health, irrespective of the variety of underlying causes and diagnoses that can be made. Thus, we considered for this study the declaration of an animal as ‘NHC’ as our end point, too.

In this study, we have analyzed the number of animals considered suitable for human consumption (SHC) after *post mortem* inspection and the number of NHC animals as related to their *ante mortem* inspection (AM) data and/or their FCI forms, and whether there was a pattern to be seen between these findings and the PM results.

Animals with no clinical findings at *ante mortem* inspection were assigned to a group that was called *Ante Mortem*-1 (AM-1). If there were remarks on *ante mortem* inspection, such animals were placed either in a group of animals showing local deformities that was called *Ante Mortem*-2 (AM-2), or in a group we called *Ante Mortem*-3 (AM-3). The latter group consisted of animals with remarks about their habitus (e.g., abnormal postures, abnormal coats, general signs of discomfort, fatigue, emaciation, etc.), but which were not considered to be sick and/or otherwise unfit to be slaughtered for human consumption. Animals that were declared unfit to be slaughtered at AM inspection were by definition excluded from the spreadsheet.

Likewise, the origin (province), parity and the breed of the animals were included in the analysis. For this, the breeds were categorized as ‘Holstein-Friesian’ (HF), ‘Meuse Rhine IJssel’ (MRIJ) or ‘other breeds’. Parity was considered as a proxy for age.

Finally, with the aid of simple 2 × 2 tables, we looked into some of the test characteristics and measures of agreement between the FCI forms and the AM results, using the PM results (NHC or SHC) as the “gold standard”. This was because these two elements of the meat inspection procedures can also be considered as diagnostic (screening) tests for sieving out animals that pose a hazard to the consumers’ health.

### 2.4. Statistical Analysis

All analyses were carried out in **R** (free software environment for statistical computing and graphics). The number of NHC cases was analyzed using logistic regression analysis with, as independent variables: province, *ante mortem* information, breed and number of calvings. To see if the *ante mortem* effect on the number of NHC depended on province, an “*ante mortem*–province interaction” was added to the model.

For similar reasons, a “breed–*ante mortem*” interaction and “number of calvings–*ante mortem* interactions” were added to the model. Akaike’s Information Criterion (AIC) was used for model reduction. For the effects that were important according to the AIC odds-ratios and their profile (log-) likelihood confidence intervals were calculated. The log odds ratio for AM-2 (only local deformities) or AM-3 (aberrant habitus) were calculated against the AM-1 group (no abnormalities at the time of AM inspection).

For the number of calvings, a Poisson model was used with province and breed as independent variables. For important effects according to the AIC, odds-ratios and their 95% profile (log-) likelihood confidence intervals were calculated. (The complete results of this analysis and the R-script used are included in the Supplementary Results section as File S1 R-output and File S2 R-script).

## 3. Results

From the in-total 223,600 slaughtered dairy cows, 212,546 originated from 9500 farms throughout the whole of the Netherlands. The remaining 11,054 animals were imported from, mainly, Belgium (ca. 80%), Germany (ca. 18%) and France (ca. 2%). When important data were missing, imported animals were excluded from the analysis. Of the 223,600 slaughtered dairy cows, a total of 3933 animals (1.8%) were considered ‘NHC’ at PM. Table 1 summarizes the findings with respect to the categorization of the animals in groups with or without certain remarks during AM inspection, as written down on the official VOS forms.

The AM results differed between categories regarding the likelihood of an animal to be declared ‘NHC’ during PM. Animals from the AM-2 and AM-3 groups were significantly more likely to become declared ‘NHC’ than animals from the AM-1 group. On average, the calculated odds-ratios for an animal to become declared ‘NHC’ were 2.99 (2.42 < OR < 4.60; 95% confidence interval, ci) for the AM-2 group, and for the AM-3 group were 4.04 (2.71 < OR < 6.26; 95% ci).

The origin, i.e., the province where the animals came from, had a small but statistically significant effect on the likelihood that animals were being declared ‘NHC’ during PM (see File S1 R-output, and Table S1 Provinces-distances-NHC in the Supplementary Materials). However, these differences were inconsistent. It appeared that not the geographical distances, per se, but other factors that we did not consider when compiling the dataset played a role in this. The cause of these inconsistent differences in outcomes of certain particular regions should be further investigated, but a plausible explanation will be given in the discussion.

The differences between the parity of the slaughtered dairy cows and the number of carcasses declared ‘NHC’ during PM is shown in Table 2. From the total number slaughtered, 20.928 animals were excluded, because they were bulls or imported animals from which the parity was unknown. Almost 80% of the slaughtered animals had only calved four times or fewer. The percentage of animals declared ‘NHC’ more or less increased incrementally with parity, with a maximum percentage of almost 11 at a parity of ten calvings (OR = 1.032; 1.015 < OR < 1.049; 95% ci). 

The number of ‘NHC’ cases was also analyzed, using logistic regression analysis, and this demonstrated that the AM effect is dependent both on the number of calvings and on the breed (included in the supplementary materials).

The differences between breeds with regard to the number of animals declared ‘NHC’ is shown in Table 3. There was a significant difference between the Holstein Friesian and the other breeds. Imported animals were excluded from the calculations because their breed was often not known.

Animals from the Holstein-Friesian breed were significantly more likely to be declared ‘NHC’ than were cows from all other breeds. Depending on whether the animal was noted to display local abnormalities (AM-2), or more generalized signs of distress (AM-3), the OR varied from 2.4 to 3.0 (2.37 < OR < 3.03; 95% ci) for AM-2 animals, and between 1.3 and 2.3 for the AM-3 animals (1.32 < OR < 2.32; 95% ci).

From the 212,546 Dutch dairy cows brought to this slaughterhouse, only 7038 (3,3%) had one or more of the relevant questions answered with ‘yes’ on their Food Chain Information forms (designated either as ‘FCI ok’ or ‘FCI Not ok’). These questions were about recent illness, the use of veterinary drugs and about withdrawal periods of the drugs used. There never was any ‘yes’ answers regarding the questions about the (disease) status of the holdings (e.g., *Salmonella*, paratuberculosis, etc.) or about relevant results from previous AM or PM inspections of animals from the same holdings. Of these 7038 animals, 380 (5%) were declared ‘NHC’ at PM. Table 4 summarizes the PM results with regard to each of the three AM categories.

If the FCI is to be seen as a diagnostic (screening) test, with the PM results regarding ‘NHC’ as the gold standard, and calculated with a simple two by two table, the overall sensitivity (5.4%) and predictive value (10.4%) regarding animals being declared ‘NHC’ on the basis of any of the relevant questions being answered with ‘yes’ will be low. On the other hand, the negative predictive value seemed high (96%). In other words, in those cases that all FCI questions were answered with ‘no’, there was a 96% probability that the animal would also not be declared ‘NHC’ at PM. However, the likelihood ratio of a positive or negative test result, which indicates whether there is an increased probability of finding or not finding an ‘NHC’ animal at PM, was 3.4 and 0.96, respectively. That points towards the FCI being not useful as a quick test for sieving out likely ‘NHC’ or ‘SHC’ animals (the calculations are included in the uploaded Supplementary Materials).

The calculations on the overall properties of the AM inspection as a diagnostic test and the PM inspection as the gold standard showed that the overall sensitivity was 56.8% and the overall specificity 99.2%. In other words, if an animal had no remarks at the *ante mortem* inspection, there was a more than 99% probability that it would not be declared ‘NHC’ at *post mortem* inspection. In addition, the ‘likelihood ratio’ of a positive test result (LR+) was 16.3 and the LR- 0.46, a clear indication that the test results indeed lead to greater probabilities of finding or not declaring an animal NHC at PM (the calculations can be found in the uploaded Supplementary Materials).

When regarded as a set of parallel (screening) tests, the sensitivity of the combination of the FCI form and the AM inspection resulted in an overall sensitivity of the combined test results of about 59% and an overall specificity of 95%, which is lower than when the specificity of each test is considered separately. However, whether or not these tests can be considered as a useful combination that improves the performance of EU inspection procedures is entirely debatable. The determination of the measure of agreement between these two tests with Cohens’ kappa showed that the agreement between the two tests was far from acceptable. The Cohen’s kappa value for agreement between the two tests regarding animals declared ‘NHC’ at PM was −0.15 and for animals declared ‘SHC’ at PM about 0.07. This means that the two tests disagreed, and apparently measure different things and cannot be seen as a useful combination (calculations shown in the uploaded Supplementary Materials as File S3 Analysis with 2 × 2 tables).

## 4. Discussion

### 4.1. General Remarks

As far as we can conclude from the literature, there is little research into the value of current official EU meat inspection procedures in culled dairy cattle with respect to an efficient protection of the consumers’ health on the basis of the data of large numbers of animals slaughtered. This study briefly looked into the relationships between the data of the Food Chain Information, AM inspection, PM inspection and the number of animals declared not fit for human consumption (‘NHC’) on the basis of a dataset that was derived from individual handwritten forms from over 223,000 slaughtered dairy cattle. The only recent study that used the data of large numbers of slaughtered bovines is a French study by Dupuy et al. in 2013 [21], which included the data of over 50,000 bovines that were slaughtered in 12 different slaughterhouses. However, that study was strictly aimed at assessing whether or not the meat inspection results as such could be used for (regional) syndrome surveillance, and did not allow for any inferences regarding the efficacy of inspection procedures as a diagnostic test used for the protection of the consumers’ health.

### 4.2. Influence of Breed, Province, Parity and Slaughterhouse

The province the animals originated from had a small but statistically significant effect on the AM and PM inspection results and numbers of animals declared ‘NHC’, but these were inconsistent with the geographical distances to the slaughterhouse (Table S1 in the uploaded extra materials). An explanation could be that there were distinct differences in travel circumstances and/or the total duration of the journey to the slaughterhouse. Given that the Netherlands is a small country with maximum travel distances well below 400 km, a better explanation is that there are differences between provinces in the ways that the slaughterhouse obtained its animals. Later inquiries, made at the slaughterhouse and with different traders, revealed that there were distinct differences between the provinces and the number of animals purchased by agents of the slaughterhouse or bought via traders, whereby the agents appear to buy animals in a somewhat better condition. However, because these data could not be included in the dataset, this needs further investigation. Normally, these data are not a part of the FCI, or registered by the NVWA.

The slaughterhouse chosen can be considered fairly representative for the health situation in the Netherlands regarding the dairy cows brought to slaughter. This is in line with the results on slaughtered pigs reported by Harbers et al. [22], who also demonstrated that, in the Netherlands, the large slaughterhouses provided for the fairest representation of the nationwide health situation in a population of slaughter animals.

Inspection data can vary from inspector to inspector and from slaughterhouse to slaughterhouse. Regarding this, the use of a single, very large slaughter facility was considered an advantage. In the slaughterhouse that provided the data for this study, meat inspection was carried out by a stable group of experienced veterinarians that have worked there together for many years in a time-pressured, high-throughput environment, thus most likely ensuring optimum and uniform performance under stress. After all, studies by Harbers et al. [22] showed that the detection of clinical signs and pathological anatomical abnormalities differ greatly between meat inspectors, and that their performance is clearly influenced by, amongst other things, their working experience and their ability to work under time pressure.

When meat inspection is considered as a diagnostic (screening) test, it seems that, at least under circumstances resembling those under which dairy cows in the Netherlands are being kept, in over 99% of cases, a favorable result of the AM (no remarks at all) also meant a favorable result of the *post mortem* meat inspection (fit for human consumption; ok). In other words, in those cases the *post mortem* meat inspection procedures could just as well have been omitted.

The differences in outcome of the meat inspection between the two largest bovine breeds in the Netherlands can be explained by differences in robustness between the Meuse-Rhine-IJssel (MRIJ) and Holstein-Friesian breeds. Meuse-Rhine-IJssel cattle are still largely dual purpose animals and are generally considered robust, fertile and with firm, sturdy legs [23,24]. Holzhauer et al. [23] and Waag et al. 2005 [24] noted distinct differences in robustness between the Holstein-Friesian and Meuse-Rhine-Ijssel breeds with regard to a number of disease conditions. Not surprisingly, the conditions that were studied by Holzhauer et al. [23] and Waag et al. [24] make up of a large percentage of the conditions mentioned on the AM forms in this study (mastitis, lameness, vaginitis and other urogenital problems). In addition, also internationally, these health conditions (fertility problems, mastitis, lameness) comprise the main reasons for culling dairy cows [25,26,27,28].

### 4.3. Use of FCI

When the FCI is considered a means for pre-selecting the more ‘risky’ cows (i.e., a diagnostic test carried out independently from the AM inspection) it does not perform as well as it potentially could or should. In fact, the results of the FCI in this study on culled dairy cows in the Netherlands seems to bear little or no significance with regard to the results of the AM or PM inspection and, in the vast majority of cases, the FCI forms were not informative at all. Of the 212,000 forms that were specifically analyzed, only 7038 (3.3%) displayed any answer to questions that were related to the health and medicinal history of the animal. In these cases, the sensitivity of the FCI information with regard to animals being declared ‘NHC’ was approximately 5%. In contrast, the sensitivity of the AM inspection was approximately 57%. Furthermore, when looking at the measure of agreement between the FCI and the AM as a diagnostic test for sieving out ‘NHC’ cows, it appeared that the results of the FCI and the AM disagreed strongly and that these had little in common. At least in our study, in the Netherlands with culled dairy cows, the current FCI information and/or the way it is being used seems to be of little added value with regard to ensuring meat safety.

Nevertheless, this does not mean that the FCI as such is a bad instrument. What it does mean is that the competent authorities have to assess whether the FCI is indeed used as intended by the farmers and slaughterhouses. For example, in our study, a mere 3.3% of all the forms were filled out by the dairy farmers with a ’yes’ on any of the relevant questions about recent illness, the use of veterinary drugs and about withdrawal periods of the drugs used. However, there were never any ‘yes’ answers regarding the relevant FCI questions about the (disease) status of the holdings the cows originated from (e.g., *Salmonella*, paratuberculosis, etc.) or about relevant results from previous AM or PM inspections of animals from the same dairy farm. From our own personal experience, Dutch dairy farmers seem to be foremost fixated on the questions about the recent use of veterinary drugs. Additionally, farmers were very reluctant to provide anyone with information that might harm their reputation or the outcome of the AM and PM, for example, the disease status of their dairy herd, or animals declared ‘NHC’ in the past. Additionally, farmers do not generally understand how many of the questions on the FCI forms relate to meat safety, possibly because meat production is not their core business and is often considered by them as an unavoidable necessity. Moreover, because the Dutch Food and Consumer Product Safety Authorities do not keep records of the herd histories, the dairy farmers are able to continue with this behavior. This aspect of the reliability of information given by farmers on the FCI forms certainly calls for further investigation.

That the percentage of ‘NHC’ animals rises with the parity (or age) of the animal was to be expected [23,24,27]. The sudden low percentage of animals declared ‘NHC’ after nine calvings in this study, however, is inexplicable and may be coincidence. The low percentage ‘NHC’ of the “very old” cows (i.e., >10 calvings) is possibly due to their already proven robustness by their long on-farm career.

### 4.4. Current AM/PM Examination, Use of FCI and Public Health

From earlier studies [29] and from the opinion on public health hazards in bovine meat of the EFSA (European Food Safety Authority), it can be inferred that, in fact, almost none of the hazards that they considered as currently important could be detected by our current AM and PM procedures [15]. However, when PM examination is conducted without any incisions (‘vision-only’), the sensitivity of the detection of cysticercosis and bovine tuberculosis will drop [8]. Nevertheless, bovine tuberculosis is, nowadays, not an important threat anymore in countries with an optimally organized animal health care system and with effective eradication programs in place in practice, which is the case in countries such as the Netherlands, Germany or Denmark [8,15]. 

With regard to cysticercosis, sarcosporidiosis and toxoplasmosis, the question arises of whether omitting PM procedures does indeed lead to major increased risks to the consumers’ health, and if there are alternative ways for preventing or mitigating any of these existing consumer health hazards. 

In Europe, the prevalence of *Cysticercus bovis* in dairy cows is generally between circa 1 and 6%, and the sensitivity of detecting cysticercosis during PM examination in general 20% (10–30%) [8]. In other words, currently about 80% of cattle that are actually positive for cysticercosis will already pass PM unhindered and thus—in general—only about 0.25–1.50% of the slaughtered animals in Europe will, at PM, be labelled as positive for *Cysticercus bovis*. Furthermore, only 10% of the cysts found in these carcasses are viable and DNA sequencing of the cysts showed that about 20% of the viable and 50% of the non-viable cysts are not *Cysticercus bovis*. The probability of an infection with *Cysticercus bovis* increases with the age of the animals and the way they were grazed and housed. Therefore, a more risk-based approach, with surveillance in combination with ELISA testing of animals at risk of an infection, would provide a far better way of detecting animals with *Cysticercus bovis* than any current standard PM inspection could ever provide [8,15,21,30,31,32,33,34].

Studies in various European countries show that, in general, 80% or more of dairy cows are carriers of sarcosporidia. Bovines are an intermediate host for several sarcosporidia species, with probably the most important ones being *Sarcocystis bovihominis* and *S. cruzi.* In Western Europe, *S. bovihominis* is the most important zoonotic sarcocystis species carried by cattle and *S. cruzi* is a non-zoonotic species, because it has dogs as the final host. *S. cruzi* is also the most common *sarcocystis* species, and is carried by up to 75% of dairy cows. Current standard PM inspection procedures will identify only macroscopic lesions, which are mostly caused by *S. cruzi* and never by *S. bovihominis*. Moreover, the role of sarcosporidia in eosinophilic myositis in cows is still unclear [35,36,37]. Therefore, omitting PM inspection procedures for each individual carcass will be of little consequence to the already existing possible health hazards for consumers of beef.

In the case of echinococcosis, PM inspection procedures also have a low sensitivity when detecting smaller hydatid cysts. The Netherlands and many other parts of Europe are not considered endemic regions for echinococcosis, and most human cases of cystic echinococcosis are caused by eating raw vegetables or berries contaminated with feces from dogs or foxes (or other carnivores). In Western European countries where echinococcosis is sporadically found, meat inspection in cattle would suffice when the lungs and livers from imported cattle from countries where echinococcosis is endemic are condemned [38].

*Toxoplasma gondii* is one of the most important foodborne pathogens. Conventional PM inspection does not detect the tissue cysts of *Toxoplasma.* Most human infections occur after the ingestion of raw vegetables contaminated with cat feces, gardening without gloves and/or improper hand hygiene, or after cleaning the cat litter box and infection by the ingestion of tissue cysts in undercooked or unfrozen meat. Sheep are more often infected than cattle, but eating undercooked or raw beef is quite common. There is no detection of toxoplasmosis during the conventional PM inspection [28]. Again, omitting PM for a large number of carcasses will be of little consequence to the already exiting consumer health hazards.

Although, according to EFSA (31), drug residues are not considered a hazard for public health related to the consumption of bovine meat, it can be a reason for condemnation of the carcass. Food chain information should be a method of pre-selecting animals with suspected drug residue risk, but because of the limited number of FCI forms with information on health and medication (3.3% of FCI forms) and the pre-selection of animals to be slaughtered (no animals with serious health problems), whole carcass condemnation related to the risk of drug residues in this dataset was minimal. Improvement of the FCI is necessary for this specific risk.

### 4.5. Risk-Based Meat Inspection

It is clear that, at present, the protection of consumer health via the pre-slaughter collection of Food Chain Information followed by an AM and PM inspection does not function as it should, and that the system needs serious improvement to work properly again. When PM inspection procedures are omitted,* a priori* knowledge of the slaughter animals becomes even more important. Regarding this, the information collected via the Food Chain Information forms should be vastly improved, because the current forms contributed practically nothing to the PM decisions that were made. Additionally, the consequences of limiting or omitting PM inspection of animals without remarks should be investigated further [16,18,31].

In a future risk-based system, the AM inspection by an official veterinarian should remain in place. The PM inspection should only be performed when an animal is a hazard for the hygiene of the slaughter line (e.g., an excessively dirty animal, or an animal with an abscess and/or a hazard for food safety/public health). This would be the case if the results of the AM inspection gave reason to suspect this, when animals are of a certain age–breed combination, when they stem from a region or herd where, in the past, more than the usual numbers of animals were declared not suitable for human consumption [21], and/or when the Food Chain Information calls for it. The FCI should, therefore, always include important known risk indicators, for example those that were identified in this study. The FCI should, or could, for example, then contain information about parity (age), breed, and region, including the endemicity of certain diseases or environmental contaminants from the region the animal comes from. Other information may include the results of serological or other tests on the presence of certain diseases that were carried out (e.g., *Toxoplasma*, STEC), the herd history regarding diseases and treatments, and reports of animals from this farm that were declared ‘NHC’ in the past. With regard to these elements, and the possible lack of compliance, Dutch dairy farmers showed that, when filling in these forms, it is worth considering complete digitalization of the FCI. Thus, all the relevant information can be automatically retrieved by the slaughterhouse and the competent authorities, totally independent from FCI-forms that have to be filled out by hand by the farmers. Finally, by continuation of the AM inspection, animal welfare monitoring and the detection of notifiable diseases can still be carried out as intended [16,18,39].

## 5. Conclusions

This study has shown that, at least as a ‘proof of concept’, slaughterhouse data about culled dairy cows can be used for determining whether or not certain elements of our current set of fixed EU meat inspection procedures could be omitted and, thus, be changed into a more risk-based approach without negative consequences for public or animal health. With regard to Dutch dairy cows that are being brought to slaughter, the AM and PM inspections have a substantial overlap. With regard to food safety and public health, in over 99% of cases the PM could even be omitted on the basis of the AM, provided our current FCI is massively improved and all the risk factors that influence the inspection findings are known. To improve the reliability of the FCI, a transition to a fully automated system is worth considering. Such a system could prevent the information being unreliable due to incomplete or misleading information on forms filled out by the (dairy) farmers themselves. 

However, what we found in our study on culled Dutch dairy cows that are being slaughtered in large scale slaughterhouses does not necessarily apply to smaller or other types of operations, other animal species, other countries or other regions throughout the EU. That is an integral part of risk-based meat inspection: for every situation, it should be determined—on the basis of identified risk factors—which elements of the meat inspection procedures should be improved or can be omitted. Thus, risk-based meat inspection will improve, in terms of the protection of public and animal health and welfare, while at the same time being as cost-effective as possible.

## Figures and Tables

**Table 1:** Overview of the number of animals slaughtered within each AM category and the number of animals that were declared ‘not suitable for human consumption’ (NHC) during PM.

AM Group	Number	NHC (%)
AM-1 (no remarks)	213,744	1700 (0.8%)
AM-2 (local abnormalities)	7195	1111 (15%)
AM-3 (aberrant habitus)	2661	1122 (42%)
Totals:	223,600	3933 (1.8%)

Note: all slaughtered animals in this table were not severely ill or otherwise unfit for slaughter; hence, it is stressed that animals with serious health problems, severe mastitis, inability to walk, severe pneumonia are NOT included.

**Table 2:** Overview of the number of slaughtered animals, categorized by number of calvings and the number of animals declared ‘not suitable for human consumption’ (‘NHC’) during PM examination.

Number of Calvings	Total Number	NHC (%)
0	22,070	141 (0.6%)
1	35,220	462 (1.3%)
2	23,7089	472 (1.1%)
3	35,222	533 (1.7%)
4	28,920	572 (2.0%)
5	19,752	467 (2.4%)
6	11,791	289 (2.5%)
7	6447	365 (5.7%)
8	3296	265 (7.8%)
9	1573	44 (2.8%)
10	813	89 (10.9%)
11 or more	539	3 (0.6%)
Total:	202,732	3933 (1.8%)

**Table 3:** Overview of the number of animals categorized by breed (HF: Holstein-Friesian; MRIJ: Meuse-Rhine-IJssel) and the number of animals that were declared ‘not suitable for human consumption’ (‘NHC’) during PM examination.

Breed	Total Number	NHC (%)
HF	145,182	2871 (2.0%)
MRIJ	60,714	718 (1.2%)
Other breeds	6650	59 (0.9%)
Total:	223,600	3648 (1.6%)

**Table 4:** Numbers of animals that had one or more questions answered with ‘yes’ on the FCI-form (i.e., FCI-Nok = FCI Not OK) and the PM results per AM inspection category (1: no remarks, 2: local abnormalities, 3: aberrant habitus; ‘SHC’ is suitable- and ‘NHC’ is not suitable for human consumption).

	AM-1		AM-2		AM-3		Totals	
FCI-Form:	SHC	NHC	SHC	NHC	SHC	NHC	SHC	NHC
FCI-Nok	6148	319	246	19	264	42	6658	380 (5.4%)
FCI-ok	198,287	1280	2531	1001	1442	987	202,240	3268 (1.6%)
Total	204,435	1599	2777	1020	1686	1029	208,898	3648 (1.7%)

## Data Availability

The dataset that was compiled is formally owned by the Netherlands Food and Consumer Product Safety Authority (NVWA) and the slaughterhouse involved. It contains many data that fall under the Dutch and European privacy legislation and cannot be distributed freely. On a case by case basis, the authors may consider sharing parts of the dataset.

## Supplementary Materials

The following supporting information can be downloaded at: https://www.mdpi.com/article/10.3390/foods12030616/s1, File S1 R-output docx.; File S2 R-script docx; Table S1 Provinces and Animals declared NHC, grouped by travel distances; File S3 Analyses with 2 × 2 tables
