# Supplementary material for: The Value of Current Ante Mortem Meat Inspection and Food Chain Information of Dairy Cows in Relation to Post Mortem Findings and the Protection of Public Health: A Case for a More Risk-Based Meat Inspection
Source: Foods. 2023 Feb 1;12(3):616. doi: 10.3390/foods12030616 (PMC9914314; doi:10.3390/foods12030616)
Supplement: Supplementary file 1 [file foods-12-00616-s001.zip › File S1-R-output.pdf]

==
==============
NHC analyse
==
==============
The number of NHC cases was analyzed using a logistic regression with independent variables:
province, antemortem, color/race and number of calvings. To see if the amtemortem effect on the number of NHC depends on province a antemortem-province interaction was added to the model. The same was done for the color/race so a color/raceantemortem interaction was added to the model and for the number of calvings. Model checking was done using deviance residuals. Akaike's information criterion was used for model reduction. For the relevant effects (according to AIC) 95% (log-)likelihood profile confidence intervals were calculated.

Uit onderstaande tabel blijkt dat alle interacties erg van belang zijn volgens AIC. Dat betekent dat er een antemortem effect (am) is maar dat t afhangt van de provincie, van kleur/ras en van pariteit.
Parity was divided int0 3 groups according to the NHC log odds ratio for antemortem class 2 and 3 versus class 1.
xgekalfd=1 pariteit= 0,1,2,3
xgekalfd=2 pariteit= 4,5,6,7
xgekalfd=3 pariteit= 8.9,10,11
Df Deviance AIC
<none> 2877.9 4272.3
factor(xgekalfd):factor(am) 6 4021.6 5404.0
factor(provincie):factor(am) 22 3245.6 4596.0
factor(kleur.ras):factor(am) 4 2906.8 4293.3
De NHC odds ratio's voor am 2 en 3 versus 1 en betrouwbaarheids intervallen per pariteitsgroep:

2.5 % 97.5 %
OdssRatio
factor(xgekalfd)[0,3]:factor(am)2 52.134275594 3.705200e+02
148.966692
factor(xgekalfd)(3,7]:factor(am)2 2.835270012 1.991664e+01
8.067592
factor(xgekalfd)(7,11]:factor(am)2 1.026146378 8.045540e+00
3.065683
factor(xgekalfd)[0,3]:factor(am)3 10.100622371 9.672419e+01
35.449534
factor(xgekalfd)(3,7]:factor(am)3 1.340586639 1.272734e+01
4.690028
factor(xgekalfd)(7,11]:factor(am)3 1.403535975 1.417347e+01
5.029336
In de pariteits groep 0 t/m 3 is de NHC oddsratio voor

antemortem 2 groep vs de antemortem 1 groep is 148.97 en het 95% (log-)likelihood profile betrouwbaarheidsinterval is (52.13;370.52). In de pariteits groep 0 t/m 3 is de NHC oddsratio voor antemortem 3 groep vs de antemortem 1 groep is 35.45 en het 95% (log-)likelihood profile betrouwbaarheidsinterval is (10.10;96.72) (4de regel tabel).
Voor de andere pariteits groepen werkt het hetzelfde.
De NHC odds ratio's voor am 2 en 3 versus 1 en betrouwbaarheids intervallen per provincie:

2.5 % 97.5 %
OddsRatio
factor(provincie)1:factor(am)2 5.213428e+01 3.705200e+02
148.96669
factor(provincie)2:factor(am)2 4.597347e+02 1.181423e+03
733.00467
factor(provincie)3:factor(am)2 1.037743e+02 2.060942e+02
146.36989
factor(provincie)4:factor(am)2 2.681724e+01 2.153954e+02
83.12863
factor(provincie)5:factor(am)2 9.194944e+01 1.601934e+02
121.54235
factor(provincie)6:factor(am)2 8.095753e+01 1.168260e+02
97.24862
factor(provincie)7:factor(am)2 3.458697e+01 8.794521e+01
55.41231
factor(provincie)8:factor(am)2 2.237291e+02 5.473836e+02
347.91418
factor(provincie)9:factor(am)2 5.848302e+01 1.824039e+02
105.45921
factor(provincie)10:factor(am)2 1.880793e+01 9.736232e+01
45.21580
factor(provincie)11:factor(am)2 2.138061e+01 7.935563e+01
42.25716
factor(provincie)12:factor(am)2 6.159458e+01 1.762891e+02
105.18128
factor(provincie)1:factor(am)3 1.010062e+01 9.672419e+01
35.44953
factor(provincie)2:factor(am)3 1.222163e+03 2.929050e+03
1872.84817
factor(provincie)3:factor(am)3 1.656102e+02 3.388014e+02
35.44953
factor(provincie)4:factor(am)3 1.671253e+01 1.650698e+02
59.27705
factor(provincie)5:factor(am)3 1.237527e+02 2.140970e+02
162.84349
factor(provincie)6:factor(am)3 2.061025e+02 3.099020e+02
252.51009
factor(provincie)7:factor(am)3 2.451194e+01 7.031363e+01
42.00885
factor(provincie)8:factor(am)3 8.233742e+02 1.968843e+03

1261.01301
factor(provincie)9:factor(am)3 1.461674e+02 3.877968e+02
2
239.32031
factor(provincie)10:factor(am)3 9.506435e+01 3.227221e+02
176.14173
factor(provincie)11:factor(am)3 1.707721e+02 3.997332e+02
258.97738
factor(provincie)12:factor(am)3 3.498063e+01 9.902752e+01
59.43406
Het werk hier net zo als bij de pariteit. Dus in provincie 1
is de NHC oddsratio voor antemortem 2 groep vs de antemortem 1
groep is 148.97 en het 95% (log-)likelihood profile
betrouwbaarheidsinterval is (52.13;370.52). In de pariteits
groep 0 t/m 3 is de NHC oddsratio voor antemortem 3 groep vs
de antemortem 1 groep is 35.45 en het 95% (log-)likelihood
profile betrouwbaarheidsinterval is (10.10;96.72). Dit is t
zelfde als voor pariteits groep 0 t/m 3 omdat de eerste regel
voor provincie 1 en pariteits groep 0 t/m 3 en het eerste ras
is.
Bij de overige provincies gaat t zelfde.
De NHC odds ratio's voor am 2 en 3 versus 1 en
betrouwbaarheids intervallen per ras:

2.5 % 97.5 % OddsRatio
factor(kleur.ras)1:factor(am)2 52.134276 370.51999 148.96669
factor(kleur.ras)2:factor(am)2 36.080938 265.49158 104.68829
factor(kleur.ras)3:factor(am)2 30.364814 309.27033 101.85486
factor(kleur.ras)1:factor(am)3 10.100622 96.72419 35.44953
factor(kleur.ras)2:factor(am)3 6.054714 59.78277 21.50923
factor(kleur.ras)3:factor(am)3 2.520726 39.62640 10.98360
>
