# Supplementary material for: The Value of Current Ante Mortem Meat Inspection and Food Chain Information of Dairy Cows in Relation to Post Mortem Findings and the Protection of Public Health: A Case for a More Risk-Based Meat Inspection
Source: Foods. 2023 Feb 1;12(3):616. doi: 10.3390/foods12030616 (PMC9914314; doi:10.3390/foods12030616)
Supplement: Supplementary file 1 [file foods-12-00616-s001.zip › File S2-R-script.pdf]

```
anmo <- read.table("antemortem.csv",sep=",",header=TRUE)

names(anmo)

[1] "provincie" "x.gekalfd" "kleur.ras" "totaal"    "SHC"       "NHC"   [7] "am1SHC"    "am1NHC"
"am2SHC"    "am2NHC"    "am3SHC"    "am3NHC"

#

attach(anmo)

anmo1 <- anmo[,c(1:4,7,8)]

anmo2 <- anmo[,c(1:4,9,10)]

anmo3 <- anmo[,c(1:4,11,12)]

#

names(anmo1)[5:6] <- c("shc","nhc")

names(anmo2)[5:6] <- c("shc","nhc")

names(anmo3)[5:6] <- c("shc","nhc")

#

am <- rep(1,length(anmo1$shc))

anmo1 <- data.frame(anmo1,am)

am <- rep(2,length(anmo2$shc))

anmo2 <- data.frame(anmo2,am)

am <- rep(3,length(anmo3$shc))

anmo3 <- data.frame(anmo3,am)

#

anmo.l <- rbind(anmo1,anmo2,anmo3)

names(anmo.l)

[1] "provincie" "x.gekalfd" "kleur.ras" "totaal"    "shc"       "nhc"

[7] "am"

#

anmo.l <- anmo.l[!(anmo.l$nhc==0&anmo.l$shc==0),]

detach(anmo)

attach(anmo.l)

table(anmo.l$nhc,anmo.l$am)

#
```

```
xgekalfd <- cut(x.gekalfd, c(0,3,7,11),include.lowest = TRUE)

fit.0 <-
glm(cbind(nhc,shc)~factor(provincie)+factor(kleur.ras)+factor(xgekalfd)+factor(am):factor(kleur.ras)+
factor(am):factor(provincie)+factor(am):factor(xgekalfd),family=binomial,data=anmo.l)

drop1(fit.0)

summary(fit.0)

cbind(exp(confint(fit.0))[17:22,],exp(coef(fit.0)[17:22]))

tapply(nhc/(nhc+shc),list(am,provincie),mean)

tapply(fitted.values(fit.0),list(provincie,am),mean)

tapply(fitted.values(fit.0),list(kleur.ras+1,am),mean)

tapply(fitted.values(fit.0),list(x.gekalfd,am),mean)

tapply(fitted.values(fit.0),list(x.gekalfd),mean)

##

fit.0 <-
glm(cbind(nhc,shc)~factor(am)+factor(provincie)+factor(kleur.ras)+x.gekalfd+factor(am):factor(provi
ncie)+factor(am):factor(kleur.ras),family=binomial)

summary(fit.0)

drop1(fit.0,test="Chisq")

exp(confint(fit.0))

detach(anmo.l)

############################################

attach(anmo)

fit.x <- glm(x.gekalfd~factor(provincie)+factor(kleur.ras),family=poisson,weight=totaal)

drop1(fit.x,test="Chisq")

summary(fit.x)

confint(fit.x)

exp(summary(fit.x)$coef[1:14])

detach(anmo)

###################################################

attach(anmo.l)

names(anmo.l)

[1] "provincie" "x.gekalfd" "kleur.ras" "totaal"    "shc"
```

```
[6] "nhc"      "am"
################################
am1s <- anmo[,c(1:4,7)]
names(am1s)[5] <-"amneg"
ns<-rep(0,432)
am1s <- cbind(am1s,ns)
################################
am1n <- anmo[,c(1:4,8)]
names(am1n)[5]<-"amneg"
ns<-rep(1,432)
am1n <- cbind(am1n,ns)
################################
am2s <- cbind(anmo[,1:4],anmo[,9]+anmo[,11])
names(am2s)[5]<-"ampos"
ns<-rep(0,432)
am2s <- cbind(am2s,ns)
################################
am2n <- cbind(anmo[,1:4],anmo[,10]+anmo[,12])
names(am2n)[5]<-"ampos"
ns<-rep(1,432)
am2n <- cbind(am2n,ns)
```
