# Supplementary material for: The Value of Current Ante Mortem Meat Inspection and Food Chain Information of Dairy Cows in Relation to Post Mortem Findings and the Protection of Public Health: A Case for a More Risk-Based Meat Inspection
Source: Foods. 2023 Feb 1;12(3):616. doi: 10.3390/foods12030616 (PMC9914314; doi:10.3390/foods12030616)
Supplement: Supplementary file 1 [file foods-12-00616-s001.zip › File S3-Analysis-with-2x2-Tables.pdf]

File S3. Analysis with 2×2 tables:

Legenda:

FCI+ is one or more questions answered with yes

FCI- is no question answered with yes

AM+ is AM-2 (local abnormalities) and AM-3 (general symptoms of distress, fatigue etc.)

AM- is AM-1 (no remarks)

PM+ is NHC (not suitable for Human Consumption)

PM- is shc (suitable for human consumption)

LR is the likelihood ratio.

LR+ means that with a positive test result the likelihood of a true positive increases.

If LR+ 1-2 the probability increases little; if LR+ 2-5 the prob increases; if LR+ 5-10 the prob increase strongly; when LR+ > 10 the probability increases severely.

LR– means that with a negative test result the likelihood of finding a true positive decreases (and finding a true negative increases).

If LR– is 1-0.5 the prob of finding a positive decreases slightly, between 0.49 and 0.20 it decreases moderately, and if the LR– 0.19-0.10 it decreases strongly and 0.09-0.01 it decreases severely (and finding a true negative increases).

FCI and AM considered separately: CAVE: THE PM RESULTS ARE THE GOLD STANDARD

FCI AS A DIAGNOSTIC TEST…

	PM+	PM-	totals
FCI+	380	3268	*3648*
FCI-	6658	202240	*208898*
totals	*7038*	*205508*	*212546*

**SENSITIVITY**	**5.40**	%	A/(A+C)
**SPECIFICITY**	**98.41**	%	D/(B+D)
**PREDICTIVE VALUE +**	**10.42**	%	A/(A+B)
**PREDICTIVE VALUE -**	**96.81**	%	D/(C+D)
**ACCURACY**	**95.33**	%	(A+D)/N
**APPARENT PREVALENCE**	**1.72**	%	(A+B)/N
**TRUE PREVALENCE**	**3.31**	%	(A+C)/N
**Likelihood Ratio pos. test result, LR+**	**3.40**		(a/(a+c))/(b/(b+d))
**LR-**	**0.96**		(c/(a+c)/(d/(b+d))

AM AS A DIAGNOSTIC TEST

	PM+	PM-	*totals*	
**AM+**	**2233**	**7623**	*9856*	
**AM-**	**1700**	**212044**	*213744*	
totals	3933	219667	*223600*	(slaughtered animals including imports)

**SENSITIVITY**	**56.78**	%	A/(A+C)
**SPECITIVITY**	**96.53**	%	D/(B+D)
**PREDICTIVE VALUE +**	**22.66**	%	A/(A+B)
**PREDICTIVE VALUE -**	**99.20**	%	D/(C+D)
**PRECISION**	**95.83**	%	(A+D)/N
**APPARENT PREVALENCE**	**4.41**	%	(A+B)/N
**TRUE PREVALENCE**	**1.76**	%	(A+C)/N
**Likelihood Ratio pos. test result, LR+**	**16.36**		(a/(a+c))/(b/(b+d))
**LR-**	**0.45**		(c/(a+c)/(d/(b+d))

	PM+	PM-	*TOTALS*
AM+	**A**	**B**	*a+b*
AM-	**C**	**D**	*c+d*
	a+c	*b+d*	*N*

In parallel testing (FCI and AM both at the same time):

1. Combined specificity: Negative (A and B) = Specificity A*Specificity B
2. Combined sensitivity: Positive (A or B) = Sensitivity A + Sensitivity B – (Sensitivity A*Sensitivity B)

In series testing (FCI first and AM thereafter, pending the pos or neg result of FCI):

1. Combined specificity: Negative (A or B) = Specificity A + Specificity B – (Specificity A*Specificity B)
2. Combined sensitivity: Positive (A and B) = Sensitivity A X Sensitivity B

	**spec**	**sens**
**paralell test**	**0.9499**	**0.5911**
**serial test**	**0.9994**	**0.0307**

FCI and AM AGREEMENT IN DETECTING/PREDICTING ANIMALS DECLARED NHC AT PM:

	FCI+	FCI-	totals
AM+	**61**	**1988**	*2049*
AM-	**319**	**1280**	*1599*
totals	*380*	*3268*	*3648*

2x2 used for evaluation of the agreement between the results of two tests:

**Observed test agreement**	**0.3676**	(a+d)/n
**Coincidental agreement**	**0.0585**	(a+b/n)*(a+c/n)
**Coincidental disagreement**	**0.3927**	(c+d/n)*(b+d/n)
**Total agreement by coincidence**	**0.4512**	is cell B8+B9
**Observed agreement minus coincidental**	**-0.0836**	is cell B7-B10
**Maximum test agreement outside the coincidental agreement**	**0.5488**	is 1- cell B10
**COHENS' KAPPA:**	**-0.1523**	is cell B20/B22

AGREEMENT BETWEEN FCI AND AM IN DETECTING/PREDICTING ANIMALS DECLARED shc AT PM:

	FCI+	FCI-	totals
AM+	510	3953	*4463*
AM-	6148	198287	*204435*
totals	*6658*	*202240*	*208898*

2x2 used for evaluation of the agreement between the results of two tests:

	**0.9516**	(a+d)/n
	**0.0007**	(a+b/n)*(a+c/n)
	**0.9474**	(c+d/n)*(b+d/n)
	**0.9481**	is cell F8+F9
	**0.0035**	is cell F7-F10
	**0.0519**	is 1-F10
**COHENS' KAPPA:**	**0.0679**	is F11/F13

	test A+	test A-	
test B+	a	b	*a+b*
test B-	c	d	*c+d*
	a+c	*b+d*	*n*

KAPPA=(observed coincidetal agreement/max test agreement outside coincidental agreement)

Kappa coefficient interpretation: values ≤ 0 no agreement; 0.01–0.20 very poor to poor; 0.21–0.40 little to fair; 0.41– 0.60 fair to moderate; 0.61-0.80 moderate to good; 0.81-0.90 good to excellent; 0.91-1.00 excellent to total agreement.

A negative kappa means that there is disagreement between the tests (worse agreement than could or should be expected).

Negative values between 0.00 and -0.1 point to "no agreement at all". Even larger negative outcomes (-0.11 and more) point at (total) disagreement, and using these two tests in combination serves no purpose.

		**results**	results	
**FCI**	**AM+** (=AM2&AM3)	**NHC**	shc	*totals*
pos	pos	61	510	*571*
pos	neg	319	6148	*6467*
neg	pos	1988	3953	*5941*
neg	neg	1280	198287	*199567*
totals		*3648*	*208898*	*212546*
