# Supplementary material for: The Value of Current Ante Mortem Meat Inspection and Food Chain Information of Dairy Cows in Relation to Post Mortem Findings and the Protection of Public Health: A Case for a More Risk-Based Meat Inspection
Source: Foods. 2023 Feb 1;12(3):616. doi: 10.3390/foods12030616 (PMC9914314; doi:10.3390/foods12030616)
Supplement: Supplementary file 1 [file foods-12-00616-s001.zip › Table S1-Provinces-NHC-distances.pdf]

Table S1 Provinces and animals declared NHC grouped by roughly estimated mean travel distances in kilometers (Noord Brabant is the province in which the slaughterhouse is located).

Province	Number	NHC (%)	Travel Distance
Noord Brabant	72845	1761 (2,4%)	50
Zeeland	6170	115 (1,9%)	100
Zuid Holland	13110	223 (1.7%)	100
Utrecht	10448	329 (3.1%)	100
Gelderland	40533	456 (1,1%)	100
Limburg	14719	243 (1,7%)	100
Noord Holland	5212	34 (0.7%)	200
Flevoland	7449	31 (0.4%)	200
Overijsel	22598	114 (0,5%)	200
Friesland	7138	113 (1,6%)	300
Drenthe	6856	65 (0,9%)	300
Groningen	5469	164 (3%)	300
Totals	*223600*	*3933 (1.8%)*	
